# Advancements in Individual Animal Identification: A Historical Perspective from Prehistoric Times to the Present

**DOI:** 10.3390/ani15172514

**Published:** 2025-08-27

**Authors:** Shiva Paudel, Tami Brown-Brandl

**Affiliations:** Department of Biological System Engineering, University of Nebraska-Lincoln, Lincoln, NE 68503, USA

**Keywords:** precision livestock farming (PLF), individual animal identification, radio frequency identification (RFID), computer vision for animal identification

## Abstract

Precision livestock farming (PLF) is rapidly advancing, with a growing array of technologies being explored and implemented to improve both productivity and animal welfare. One of the major challenges in this field is the identification of individual animals. Despite numerous efforts having been made to automate this process, there remains a lack of holistic reviews that comprehensively integrate and evaluate these technological developments. Historically, humans have employed various techniques to identify individual animals. This article provides an overview of the evolution of animal identification methods, highlighting significant transitions across various time periods. In prehistoric times, identification relied solely on visual inspection. Today, advanced methods are being utilized, such as radio frequency identification (RFID), computer vision-based systems, biometric recognition, and DNA profiling. Each identification method has its own strengths and limitations. Interestingly, early methods such as visual inspection and drawing can still inspire the development of novel automated systems when combined with modern technologies.

## Simple Summary

Widespread adoption of precision livestock farming (PLF) and technical revolution in animal farming is hindered by the inability to automatically identify individuals in farms. Humans have been growing animals since prehistoric times and have been identifying individuals; however, automatic identification is not yet widespread. The effectiveness of PLF technology, such as weight monitoring, disease detection, and feed management, has not yet been implemented to their full potential as their implementation majorly depends on individual identification. This literature review summarizes the historical development of identification from the earliest methods to modern technology.

## 1. Introduction

Humans have practiced agriculture for millennia. As part of this transition, animals were domesticated alongside crops to provide a continuous and reliable supply of food. From our earliest origins, animal protein has played a vital role in humanity’s survival and development [1,2]. Beyond providing meat, animals have contributed milk, eggs, fiber, transportation, draft power, and manure to sustain communities and agricultural systems. Over time, animal agriculture evolved from subsistence-based systems, where nearly every family kept a small number of animals for household needs, to highly specialized production systems focused on efficiency, output, and uniformity. Although animal protein has long been essential to human survival, its production methods have not evolved as dramatically as many other manufacturing industries [3,4,5]. In response to growing demand, animal agriculture has become increasingly intensified, with larger herds and flocks managed in more complex production environments, creating a need for automated systems to identify, monitor, and manage individual animals efficiently. With rapid technological advancements, it is essential to incorporate innovations into agriculture and animal farming. Precision livestock farming is a modern, automated solution that offers an ever-expanding range of technologies designed to monitor animal behavior, health, and environmental conditions in real-time, aiding animal caretakers in effective management. These systems promise to enhance productivity, improve animal welfare, reduce resource use, and support data-driven decision-making across livestock operations [6,7]. In swine production, advanced technologies are increasingly utilized for feeding, health monitoring, reproductive management, environmental control, production oversight, waste management, genetic management, and animal identification. Humans have practiced agriculture for millennia. As part of this transition, animals were domesticated alongside crops to provide a continuous and reliable supply of food. From our earliest origins, animal protein has played a vital role in humanity’s survival and development [1,2]. Beyond providing meat, animals have contributed milk, eggs, fiber, transportation, draft power, and manure to sustain communities and agricultural systems. Over time, animal agriculture evolved from subsistence-based systems, where nearly every family kept a small number of animals for household needs, to highly specialized production systems focused on efficiency, output, and uniformity. Although animal protein has long been essential to human survival, its production methods have not evolved as dramatically as many other manufacturing industries [3,4,5]. In response to growing demand, animal agriculture has become increasingly intensified, with larger herds and flocks managed in more complex production environments, creating a need for automated systems to identify, monitor, and manage individual animals efficiently. With rapid technological advancements, it is essential to incorporate innovations into agriculture and animal farming. Precision livestock farming is a modern, automated solution that offers an ever-expanding range of technologies designed to monitor animal behavior, health, and environmental conditions in real-time, aiding animal caretakers in effective management. These systems promise to enhance productivity, improve animal welfare, reduce resource use, and support data-driven decision-making across livestock operations [6,7]. Advanced technologies in animal agriculture are increasingly utilized for feeding, health monitoring, reproductive management, environmental control, production oversight, waste management, genetic management, and animal identification.

Precision Livestock Farming (PLF) incorporates a variety of sensors, including digital and depth cameras, inertial measurement units (accelerometers, gyroscopes), and microphones alongside modern computing and internet connectivity. These tools, combined with environmental and climate control systems, offer the promise of a highly effective integrated solution that will help producers optimize resources [8]. However, introducing these sensors and technologies on a farm requires robust digital platforms to interpret raw data and deliver synthesized, actionable insights. While several commercial providers offer such platforms, most currently focus on a single system, for example, feeding, climate control, health monitoring, or weight tracking rather than offering a comprehensive, integrated solution.

Animal production is transitioning from traditional systems, where animals were managed as a group and received the same care regardless of individual needs, toward more complex, data-driven approaches enabled by Precision Livestock Farming (PLF). Fully realized PLF technologies will enable producers to continuously monitor each animal and make informed management decisions based on its individual needs, ultimately improving outcomes for both animals and operations. PLF technologies create opportunities to move beyond herd-level care, but a key challenge remains: the shift from managing animals collectively to providing individualized attention. Each animal is biologically and behaviorally unique, requiring personalized care to reach its full potential. To enable this transition, robust and scalable systems for individual animal identification are essential, serving as the foundation for precise monitoring, informed decision-making, and targeted intervention.

## 2. Early Methods

**Figure 1:**
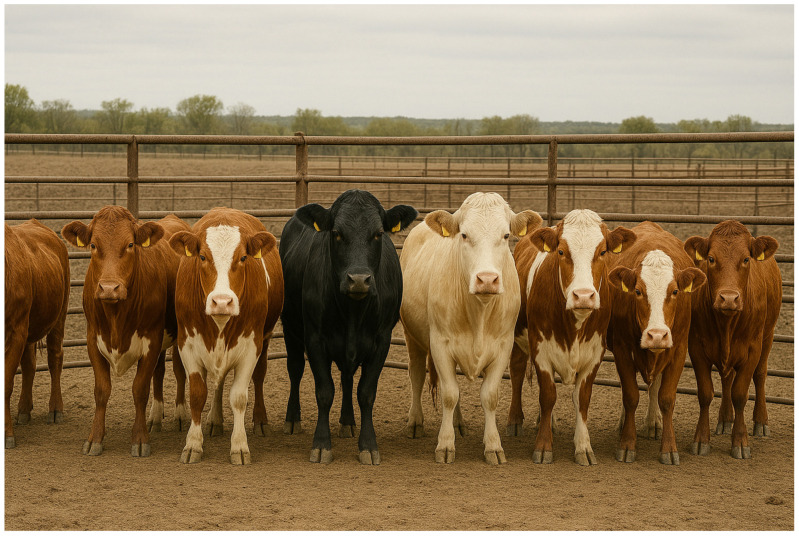
Identification based on natural markings. Differences in body shape, coat color patterns, and the size of body parts were used to distinguish individual animals.

In prehistoric times, long before written records or formal farming systems, livestock owners identified animals using natural physical traits, such as coat color, horn shape, body size, or distinctive markings (Figure 1). Evidence for this early form of animal recognition comes from Paleolithic cave paintings, like those found at Lascaux and Chauvet caves in France, which date back as far as 30,000 to 17,000 years ago. These paintings often show animals with striking individual features, suggesting [9].

By around 3000 BCE, more symbolic and standardized methods of animal identification began to emerge in the ancient Near East. Clay tablets from Mesopotamia depict animals, particularly cattle, marked with unique symbols to indicate ownership, track movement, and facilitate trade. In addition to body markings, some animals were identified using incised or painted symbols on their horns, a practice documented in both textual and archeological sources [10]. This development marks one of the earliest known efforts to formalize livestock identification as part of broader record-keeping and accountability systems.

**Figure 2:**
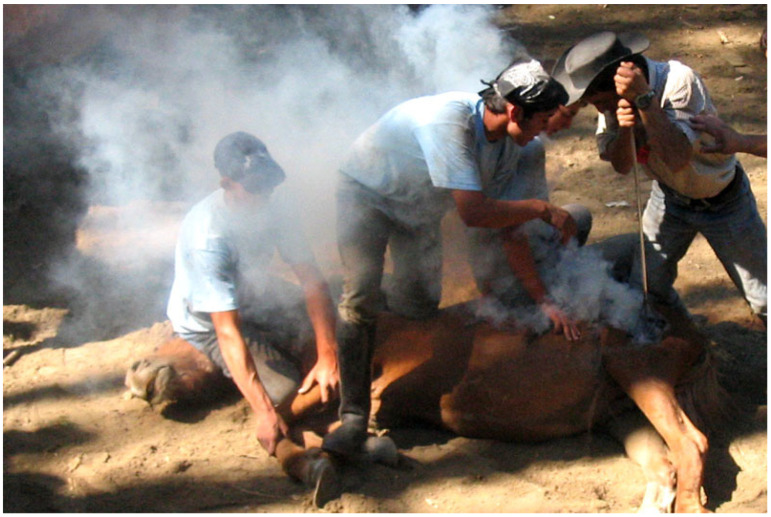
Hot metal Branding: hot metal is stamped to animals’ body to make permanent marks, image adapted from [12]. Copyright: Photo by Amio Cajander, licensed under CC BY-SA 2.0, via Wikimedia.

By 2000 BCE, hot-iron branding had become a common practice in ancient Egypt and Mesopotamia. Heated metal tools were pressed against the skin to create permanent symbols or letters unique to each owner. This durable method of identification was especially important for valuable animals such as horses and cattle. Branding persisted through the centuries, with notable examples, such as the branding of swans belonging to the Kings of England as early as the 13th Century. Figure 2 shows hot branding. In this method, red-hot metal is pressed against the animal’s skin to leave permanent marks for identification. Despite the development of alternative methods, hot-iron branding remains in use today in some regions and sectors, particularly in extensive livestock systems where long-distance ownership verification is still necessary [11].

**Figure 3:**
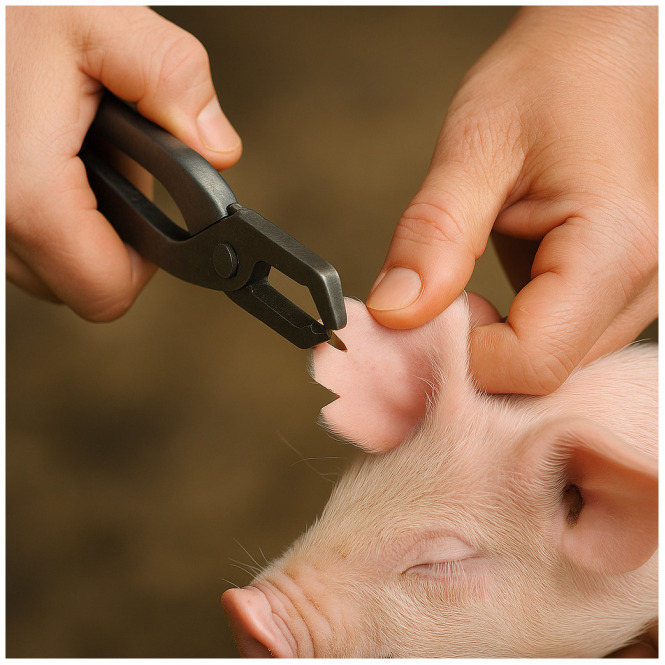
Ear Notching: animals’ ears were cut in a certain pattern, leaving a permanent marker for future identification.

Another method believed to have emerged as early as 2000 BCE was ear notching, in which specific patterns were cut into the ears of livestock (Figure 3). These notches acted as permanent, tamper-resistant identifiers that conveyed information about ownership, lineage, or birth order, and were especially useful for animals kept in larger groups. Due to their simplicity, low cost, and effectiveness, ear notching has persisted into modern times, particularly in swine production, where standardized notch patterns are still used to identify litters and individual animals.

The importance of these practices was later codified in legal frameworks, such as the Code of Hammurabi [13,14], which included laws specifically addressing animal theft and the use of physical markings for identification, further underscoring the societal value of animal traceability [10].

Overall, historical evidence shows that livestock identification has deep roots, with methods evolving from simple body markings to more sophisticated approaches like branding, reflecting the growing importance of traceability in animal husbandry throughout history. Gradual development in identification practice led to the attachment of foreign particles such as rings and tags. 1595 has the earliest evidence of using foreign particles attached to animals for identification; in 1595 Henry IV banded a Peregrine bird with a metal ring. The first recorded use of a band for identification in North America dates to 1803 [15].

**Figure 4:**
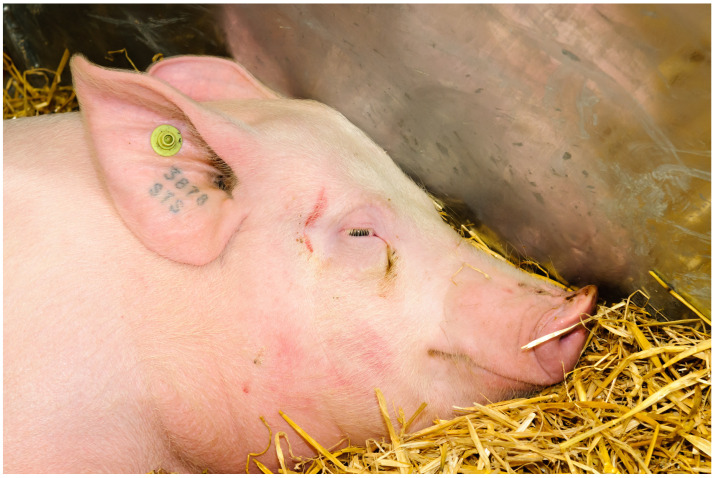
Tattooing is used as a method of permanent identification. Animals are marked with colored ink, typically applied to the inner ear, to create long-lasting and tamper-resistant identifiers. (Image © Adobe Inc., used under Education License).

Tattooing emerged in the early 20th century as a practical alternative to branding and notching, particularly for use in breed registration and individual identification. Unlike branding, which leaves an external mark, tattooing involves imprinting numbers or symbols into the animal’s skin—typically inside the ear—using a specialized plier with ink-coated needles (Figure 4). This method was favored for its relative simplicity, reduced visibility, and permanence. Tattooing became especially popular in registered purebred livestock, such as dairy cattle, sheep, rabbits, and swine, where maintaining accurate pedigree records was essential. By the 1920s, major breed associations in North America and Europe had incorporated tattooing into their official protocols for animal identification. Although it lacks visual readability from a distance, tattooing is still used today in certain sectors for permanent identification of animals, often in conjunction with more modern methods such as RFID or ear tags for enhanced management efficiency.

**Figure 5:**
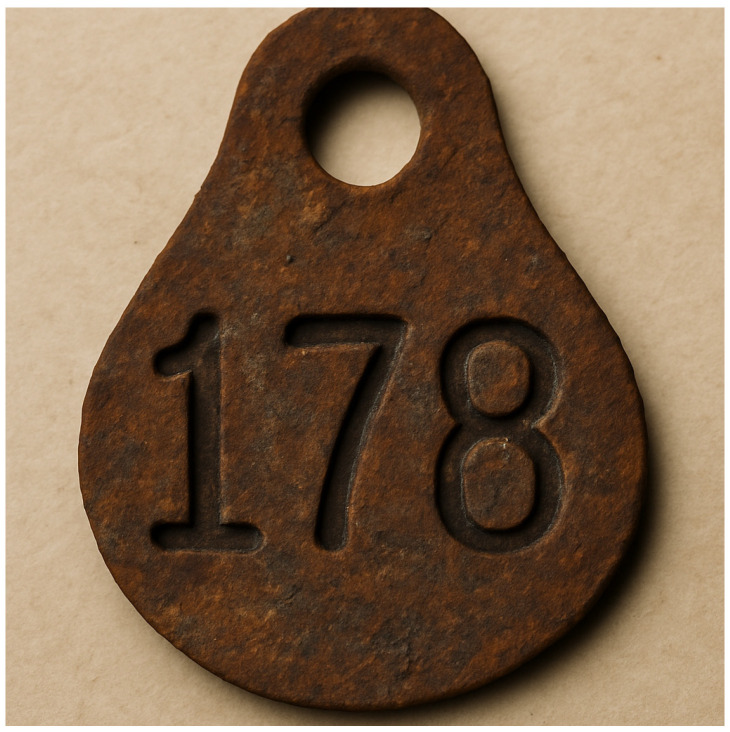
Metal ear tag used to identify animals within the herd.

With the growth in herd size and sophistication in livestock farming, the identification of farming animals evolved. However, it is unclear when exactly the first ear tags were used in animals for identification, but the first scientific evidence dates to 1799. In 1799 Sir Joshep Banks developed ear tags for King George III’s Merino sheep flock [16]. In the United States ear tags were incorporated as breed identification in 1895 with the forming of the international Ohio Improves Chester Association. The first developed tags were made of leather and quickly improved to use of metal (Figure 5). The following figure summarizes the development of an early method of identification.

**Figure 6:**
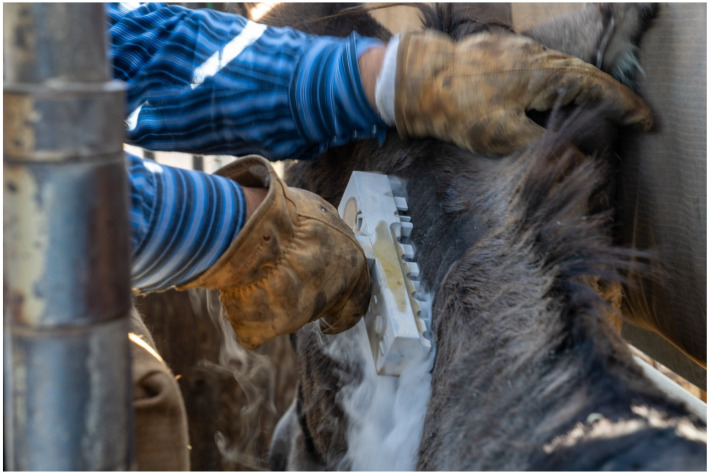
MFreeze Branding, extremely cold object in certain shape is placed in contact with an animal’s body to alter hair follicle color leaving permeant marker for future identification. Photo by Bureau of Land Management, licensed under CC BY 2.0, via Wikimedia.

During the early to mid-20th century, freeze branding was introduced as a humane alternative to hot-iron branding. This technique uses extreme cold—typically from liquid nitrogen or dry ice and alcohol—to destroy pigment-producing hair cells in the skin. As the hair grows back, it appears white, creating a visible mark without burning or scarring the animal (Figure 6). Freeze branding proved to be less painful and more animal welfare–friendly than traditional branding and gained popularity in certain species, particularly horses and cattle. It is especially effective on dark-coated animals, where the contrast makes the brand easier to read. While it requires slightly more time and specialized equipment than hot branding, freeze branding remains in use today, particularly for permanent identification in registered herds and ownership verification in extensive grazing systems.

## 3. Modern Methods

**Figure 7:**
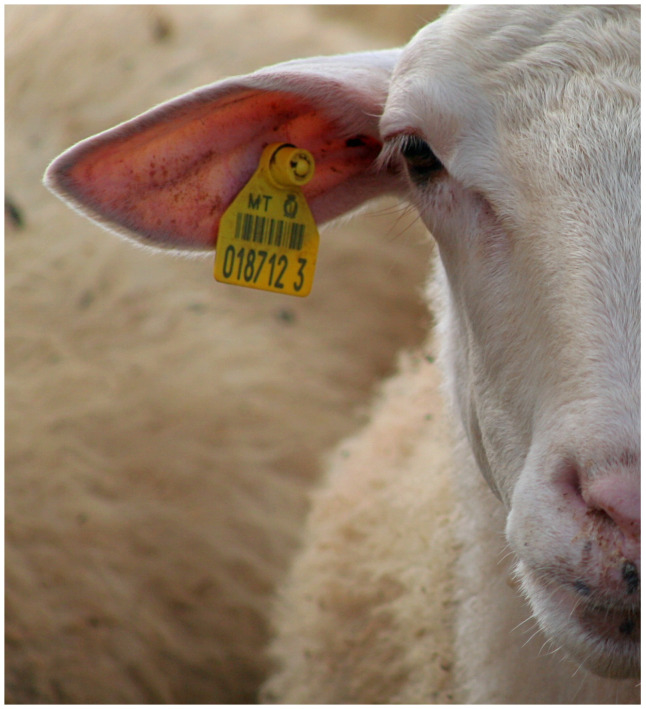
Modern-day plastic ear tags, which can be placed in the ear as early as birth and can remain in the ear through the animal’s life [20]. Photo by Continentaleurope, licensed under CC BY 2.0, via Wikimedia Commons.

Plastic ear tags are a widely adopted method for individual animal identification across livestock species, particularly in cattle, pigs, and sheep. Typically applied shortly after birth, these tags are designed to remain in place for the duration of the animal’s life, offering a practical and cost-effective solution for herd management. Made of durable plastic materials and available in a variety of shapes and sizes, they often include a unique numerical or alphanumerical code and may incorporate barcodes. Numerous manufacturers produce these tags to meet varying industry and regulatory requirements, with some designed for visual identification and others equipped with RFID technology to support automated data capture (Figure 7).

Today, animal identification technologies include RFID, biometric methods like retinal scanning, and computer vision techniques. These methods support various applications, from livestock management and disease control to biodiversity conservation and taxonomy [14,17,18]. RFID has become a standard in many countries for livestock management, aiding in traceability and disease outbreak prevention [14,19].

In the early to mid-1900s, biometric identification methods such as nose printing and iris scanning began to be used for reliable animal recognition. By the 1970s, radio frequency identification (RFID) tags and implantable microchips emerged, offering more precise and digital tracking capabilities. The 1980s saw the advent of DNA profiling, which enabled the use of genetic markers for identifying individual animals, particularly in breeding and conservation contexts. In more recent years, identification has become increasingly automated through camera-based systems. These systems utilize image processing and deep learning techniques to analyze features such as facial characteristics, coat color patterns, and 3D spatial features. Technologies like YOLO and ResNet allow for continuous tracking, while deep convolutional neural networks (CNNs) are trained to recognize animals based on visual patterns. Additionally, automatic tag reading using cameras and 3D imaging with depth sensors or point clouds further enhance the precision and automation of animal identification.

### 3.1. RFID (Radio Frequency Identification)

**Figure 8:**
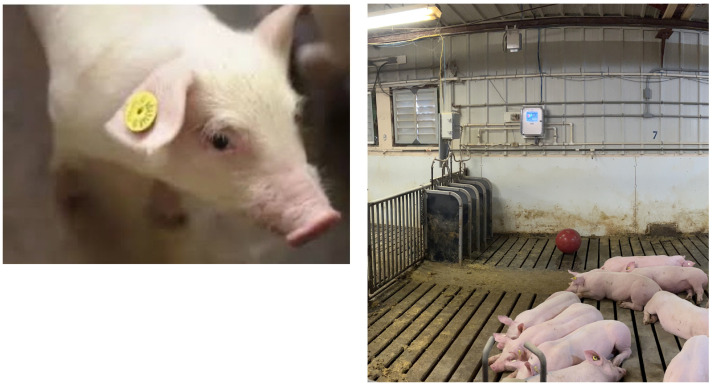
RFID ear tag and reading system. Animals were tagged with RFID chip, when the chip comes within the antennas field animals were identified by unique identifier associated with the chip. (Image courtesy of USDA Meat Animal Research Center).

With the Industrial Revolution and rapid technological development, people began applying new advancements to agriculture, including farming and livestock management. Among these innovations, RFID technology was simple and effective for livestock applications. Even today, RFID is the most used automatic identification technique (Figure 8). This study will go into detail about how the RFID technique gained prominence and recent developments in the RFID system.

The introduction of an RFID-based communication system in 1948 laid the groundwork for further exploration of its applications. From the 1950s to the 1970s, RFID techniques underwent significant theoretical progress, and in 1973, passive RFID tags were first employed for animal identification [21,22]. This marked the beginning of RFID technology’s integration into livestock farming, enabling more effective identification, tracking, and management of animals.

RFID is a broad term that refers to technologies that use radio waves to automatically identify people or objects. A standard RFID system is composed of three essential components: an RFID tag or device, a tag reader equipped with an antenna and transceiver, and a host system or a connection to an enterprise system. Together, these components enable seamless and efficient identification and tracking across various applications.

RFID tags are categorized into two types based on power sources: active and passive. Active RFID tags include a battery and can actively transmit signals to the reader, enabling identification at any time. Passive RFID tags, on the other hand, lack a power source and rely on the energy emitted by the reader’s field to function, making identification possible only within a certain range of proximity. Passive RFID tags are widely used in animal identification due to their simplicity, durability, and cost-effectiveness. They do not require an active power source, have an unlimited lifetime, and can be made as small as a sheet of paper, making them versatile for various applications. These tags contain a microchip and a circuit that draws power from the reader’s electromagnetic field to modulate a signal, enabling the reader to identify the tag.

Active as well as passive RFID tags usually have memory and can be written to with information such as identifier numbers. Writing information to a passive RFID tag requires placing the tag within the field of a reader/writer, sending a write initialization signal, and specifying where the data should be stored. Once the process begins, the system can write text, numbers, or unique identifiers onto the tag. This is particularly useful for applications like animal identification, where unique identifiers play a crucial role in distinguishing individual animals [22].

The frequency of the electromagnetic wave used by an RFID system determines critical factors such as the read range, medium compatibility, and data capacity [21]. Different frequency bands serve various applications, and their characteristics are summarized below:

When it comes to livestock, RFID tags are chosen based on the species [23], the environment, and the frequency band most suited to the application. Table 1 and Table 2 summarize criteria for choosing RFID types. LF technology is commonly used for livestock due to its compatibility with the environmental conditions in farms, such as water and metal interference. Large animals, including bovines, equines, ovine, porcine, and caprine, are often tagged with RFID ear tags. While effective, ear tagging involves puncturing holes in the animal’s ears, which can cause pain and stress. As an alternative, RFID tags integrated into neck collars are often used for dairy cattle and equines to reduce discomfort.

The placement of antennas is critical for effective RFID systems in livestock facilities. Antennas are strategically installed in areas frequented by animals, such as feedlots, water stations, or enrichment areas, to ensure reliable identification during regular activities. In setups requiring simultaneous readings from multiple animals, multiple antennas can be connected through a multiplexer.

Overall, RFID systems have revolutionized livestock management by improving the efficiency and accuracy of animal identification and tracking. The selection of frequency bands, tag types, and system configurations depends on the specific needs of the application (Table 1 and Table 2), with passive RFID technology being a popular choice for its simplicity, durability, and cost-effectiveness in animal-related applications.

While it is common to use ear tags or RFID ear tags in other species, their use is not widespread in the production of swine. Identification systems, whether RFID-based or otherwise, are primarily utilized for gestation sows, which represent only a small segment of the swine production landscape.

**Figure 9:**
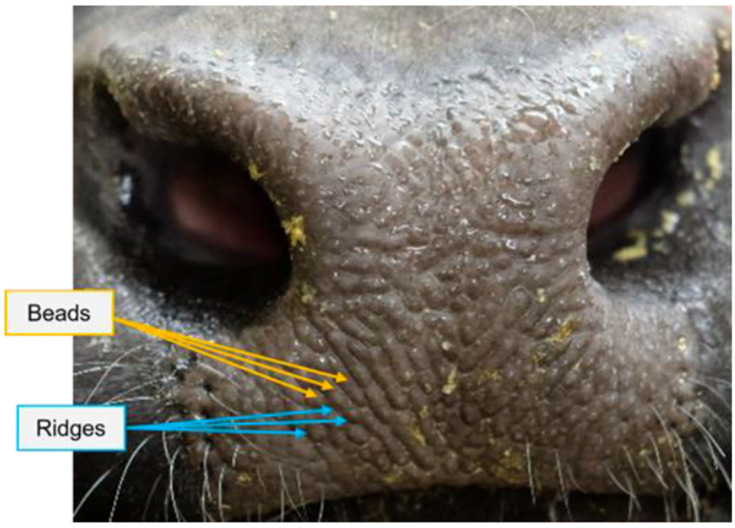
Individual identification by looking at the muzzle pattern. In initial days, muzzle patterns were printed on paper; later, vision-based techniques were used [27]. Image from [27] under CC BY license.

Biometric identification as well as DNA profiling could achieve high accuracy identification, but the process associated with these methos are not applicable to quick repetitive identification. Biometric identification works by comparing biometric information individual such as muzzle print by printing them in paper or by using camera-based techniques as shown in Figure 9 [24,25]. For DNA profiling, individuals need to be analyzes making it very laborious as well as resource consuming.

Swine production poses several challenges for RFID-based identification. First, the swine are raised in large herds within confined spaces, making individual tagging labor intensive. Additionally, they remain in production facilities for only short periods, limiting the cost-effectiveness of using tags, which are typically assigned to a single animal [26]. Their highly social behavior and difficulty in isolation further complicate the tagging process. Identification using DNA profiling can be performed for a large number of animals; however, it is not applicable to identify individuals on a daily basis or multiple times as a PLF tool.

As a result, unless simpler, more cost-effective identification methods that require minimal labor and handling are developed, identifying individual swine in commercial farming will continue to present significant challenges. One such simpler identification could be identifying animals using cameras, where individual animals do not need to be tagged or marked.

### 3.2. Camera-Based Automatic Identification

**Figure 10:**
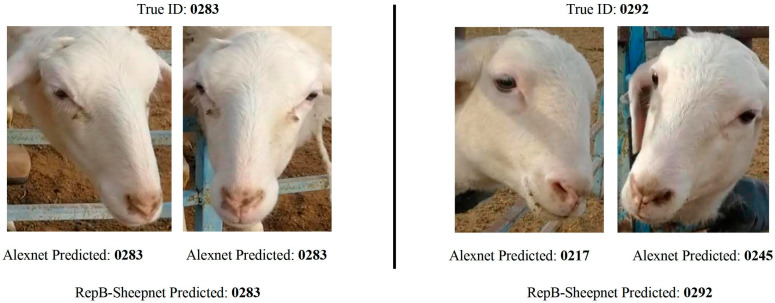
Animals’ facial images were analyzed to identify individuals. Facial features such as relative position of eyes, nose snout, etc., were used for identification [29]. Images from [29] under CC BY license.

Using cameras and vision-based identification can simplify animal identification, eliminating the need to isolate animals and tag their ears—a process that often causes significant pain. In humans, methods like facial recognition are widely used for individual identification and could serve as a more humane alternative to RFID tagging for animals. However, capturing facial images of animals is less common, and different species require customized identification approaches. Figure 10 shows use of facial image for identifying individuals in swine. A similar approach might not be efficient for identification of other species. For example, cattle and sheep often exhibit coat color variations, horses can be identified by bony protrusions, and swine shows minimal differences among individuals, necessitating the use of additional features for accurate identification [28].

The use of cameras for identification varies depending on the species. Some species have seen great use of machine vision for identification whereas some species lag in studies to adopt cameras for identification. Cameras are widely used to identify dairy cattle, mainly with modern machine learning and deep learning methods [30,31].

To summarize the broad spectrum of machine learning techniques employed in animal identification, particularly in image-based approaches, commonly used algorithms include Support Vector Machines (SVM), K-Nearest Neighbors (KNN), and Artificial Neural Networks (ANN). These methods are machine learning architectures that transform data into higher dimensions for function approximation. SVMs map data into higher-dimensional spaces to construct a hyperplane that separates different data categories. KNN classifies data by clustering it into classes based on the minimal distance to the test data. ANNs approximate higher-order transfer functions between data and labels.

Deep Learning (DL), a subset of machine learning, has gained significant popularity in recent years. An ANN model with more than two layers, including at least one hidden layer, qualifies as a deep learning model. Deep learning models can incorporate various advanced machine vision techniques, such as convolution, feature aggregation, regularization, and more, to enhance their functionality and accuracy. Notable deep learning architectures applied include Convolutional Neural Networks (CNNs) such as AlexNet, ResNet, and VGGNet, as well as object detection models like YOLO (You Only Look Once), which enable real-time identification and localization of individual animals within images.

Various anatomical features of animals have been explored for individual identification, each offering unique advantages. Among these, facial images (Figure 10) and muzzle print patterns (Figure 9) are the most used techniques [25,32,33]. Either facial images or muzzle patterns are used to train machine learning algorithms which are then later used for real time identification [32]. Building on these advancements, facial recognition techniques have also been explored for animal identification [34]. For instance, facial identification has proven effective for cattle, sheep, and goats [35] and is also limitedly adopted for swine identification as well [36,37,38,39]. This technique works by identifying stable landmarks in the facial structure, such as the relative positions of the eyes, the tip of the nose in relation to the eyes, and other features similar to those used for human identification. These relative features are extracted by use of CNN and other techniques [35]. Various models have been developed to facilitate identification based on facial images [18,40].

**Figure 11:**
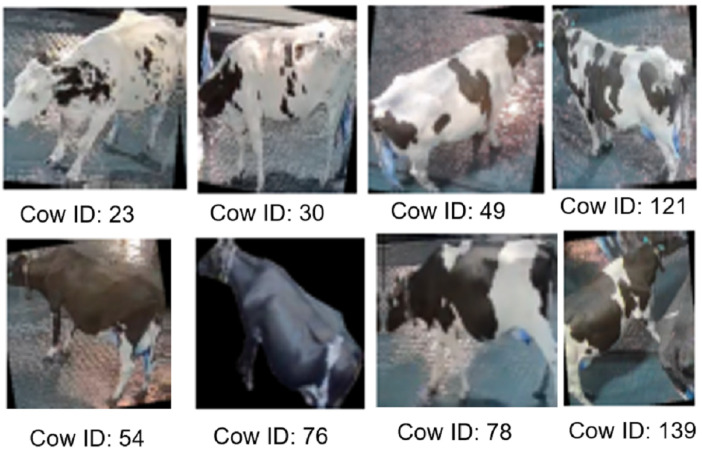
Identification based on coat color variation in the animal’s body. Images of various dairy cows’ bodies were used to train a machine learning algorithm to distinguish between individuals from [43]. under CC BY 4.0 license.

Following these, body photographs, (Figure 11) which include coat color patterns combined with physical shape and curvature, are also widely utilized [41,42]. Similarly to facial recognition, modern deep learning architectures are used to classify individuals based on body features. Facial or body recognition in bovines, sheep, and horses holds significant potential, as stable landmarks like color patterns and bony protrusions enable reliable re-identification [41]. However, facial recognition is not well-suited for swine species due to their minimal coat color variation and downward-facing body posture.

In less frequent cases, advanced modalities such as 3D imaging and retinal scans have been employed [44,45]. However, capturing retinal images in farm animals poses challenges due to their typical downward-facing posture and the safety risks of close camera placement. As a result, body image-based identification emerges as a more practical and effective approach when using cameras.

**Figure 12:**
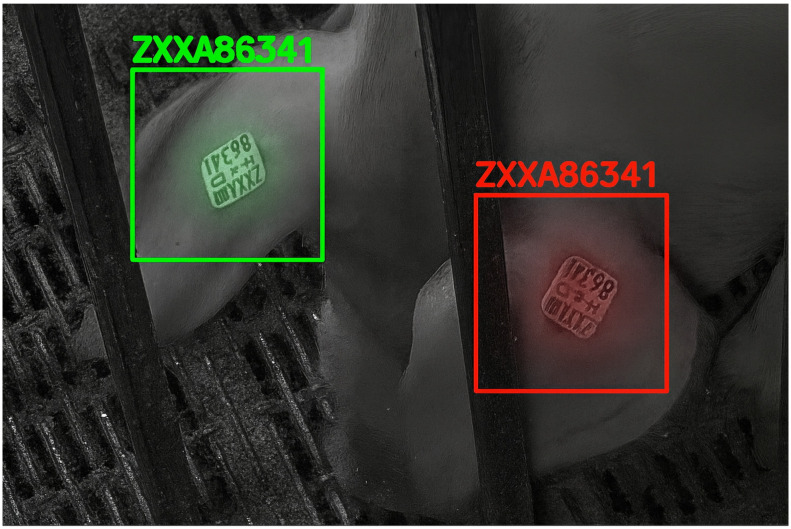
Animals were tagged with ear tagged with marking. The markings were identified by camera to identify individuals with tags. (Image courtesy of PIC, Inc., Hendersonville, TN, USA).

These deep learning-based methods can also be extended to support automated reading of small tags attached to animals, such as ear tags. As illustrated in Figure 12, modern computer vision techniques are capable of recognizing tag numbers even under challenging conditions, including partial occlusion [46]. Tag-based identification remains a reliable approach; however, it necessitates the physical tagging of animals, which may not always be feasible or desirable. As an alternative, biometric features can serve as non-invasive identifiers.

**Figure 13:**
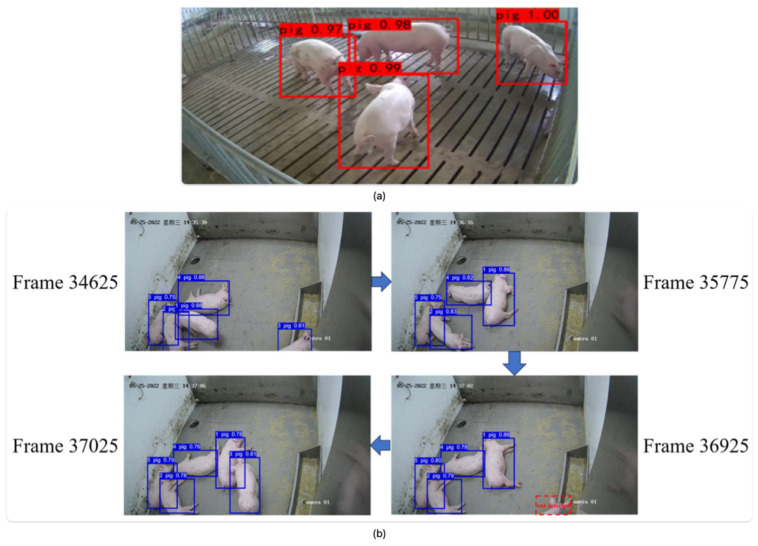
Animals were detected (**a**) [47] and continually tracked (**b**) throughout the camera’s field of view, keeping their identity preserved all the time [40]. (Images from [40,47] under CC BY license).

Additionally, modern deep learning architectures have demonstrated significant efficacy in detecting animals within image frames (Figure 13a), enabling long-term tracking while preserving individual identities (Figure 13b) [47]. Continuous monitoring of animals through these methods also facilitates effective re-identification, as supported by recent studies [48,49,50]. Although scalability to large populations remains a challenge, these approaches offer valuable opportunities for integrating vision-based predictive models to assess various aspects of animal welfare, including health status [51], behavioral patterns [28], and growth trajectories.

**Figure 14:**
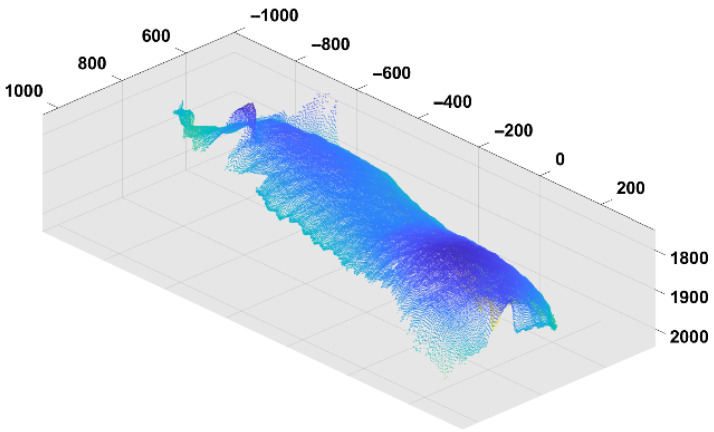
Identification by looking at 3D body features of animals. This method is most suitable for species without coat color variations such as pigs.

Some studies have also explored animal identification based on body shape, using techniques such as top-down images and point clouds (Figure 14) [52]. These approaches analyze body shape by training deep learning algorithms on images of the animals’ bodies [30]. Body shape-based identification is often more practical for farm animals, especially for swine species, as capturing high-quality facial images while maintaining a functional camera and sensing system can be a challenging task.

Recent advancements in deep learning and computer vision have significantly improved the reliability of camera-based animal identification systems. Increasingly sophisticated models—such as Vision Transformers [53] and diffusion-based architectures [54]—are being explored and implemented in this domain. By leveraging multiple biometric features, including facial landmarks, body morphology, and coat patterns, and integrating them with robust machine learning algorithms, researchers are developing highly accurate and scalable solutions for farm animal identification [55].

Although parameters such as the number of individuals used for identification, the methods employed, and the species studied vary significantly across research efforts, some general trends can be observed. Notably, 3D camera systems tend to outperform 2D systems for long-term individual animal identification, particularly in scenarios where animals undergo growth, coat changes, or are exposed to variable lighting conditions. [30,56]. While 2D systems are more cost-effective and easier to deploy, they require more frequent retraining and are sensitive to environmental factors [41,57].

Future research should focus on enhancing the adaptability of these models across different breeds, and ages, improving image capture techniques in uncontrolled farm environments, and combining multiple modalities, such as 3D and 2D imaging to increase accuracy and robustness. As technology progresses, camera-based identification has the potential to become a viable alternative to RFID, offering a non-invasive, scalable, and cost-effective solution for livestock management.

## 4. Used Cases, Feasibility and Resource Needed

The most commonly used method for individual identification among livestock is RFID-based identification. The small chip size and relatively simple setup of RFID systems offer advantages over camera-based systems. On average, RFID tags cost approximately $2–3 (US dollars) per tag, and readers range in cost from $500 to $2000 US dollars for a handheld reader to several thousand for a complete system [26]. The use of single-use RFID tags is expensive for large herds of animals and requires replacement with each new group of animals. The benefit of the RFID tag is the physical identification of an individual animal regardless of location. time compared to vision-based systems.

Camera systems, in contrast, are typically durable and do not require replacement with each production cycle. Nevertheless, implementing camera-based identification systems involves additional requirements, including specialized software and high-performance computing hardware. While RFID tagging involves a brief physical tagging process, vision-based systems may require initial data collection and model training. However, biometric identification methods—such as facial recognition—can often be developed using relatively small datasets, especially when leveraging pre-trained models or transfer learning techniques, making them a viable and scalable alternative.

Given the broader technical requirements of camera-based identification systems, there are currently only a limited number of commercial products available. However, with the increasing accessibility of advanced technologies—particularly the widespread availability of powerful mobile devices—there has been gradual progress in the development of identification systems. Some of the existing products and systems for livestock identification and monitoring include solutions such as 406 Bovine, Feed King EverAg, BETSY OneCup AI, etc.

## 5. Ethical Considerations

The use of biometric and sensor-based technologies for animal identification introduces several important ethical and logistical considerations. One key concern is data privacy and ownership. As biometric and behavioral data are collected from individual animals, questions arise regarding who owns this data—the producer, the technology provider, or a third party [58]. This issue is particularly critical as commercial platforms increasingly aggregate data across farms, raising concerns about data sovereignty and fairness, especially for independent producers. Data security is another major concern [59]. For example, the use of video cameras in barns introduces the risk that systems could be hacked, allowing unauthorized parties to access footage. Producers have expressed fear that an “isolated incident captured on video” or a “video taken out of context” could be leaked to social media and cause significant reputational harm, even when animal care is within acceptable welfare standards [60].

In parallel, RFID technology also presents ethical challenges. In many countries, RFID tags are registered in national databases, enabling comprehensive traceability throughout an animal’s life. However, the United States has struggled to mandate a national system due to producer resistance to public data-sharing—often due to concerns over privacy and regulatory control [61,62].

Finally, continuous monitoring invites deeper reflection on the role of surveillance in livestock production and the evolving human–animal relationship in technology-driven systems. Given that technological innovation often outpaces regulation, there is a pressing need for industry-wide standards and ethical frameworks that address data use, sharing, and security. Ethical data governance requires transparent policies, clearly defined producer-level agreements, and informed consent from animal caretakers when video data is collected and used. These practices are essential to ensuring the responsible use of monitoring technologies in livestock production systems.

## 6. Conclusions

Identifying individuals has always been important for livestock farming since prehistoric times. The earlies evidence of identification shows that animals were marked with clay or drawing individuals with clay. These methods gradually build up to making permanent markers either by using extreme heat or cold. Use of foreign objects attached to the animal’s body further build up to ear tags and RFID tags. The pain and labor associated with tagging the animals led to the development of camera based noninvasive automatic identification. Generally, deep learning to learn on image features was used for computer vision-based identification. Several ideas have been put forward on identification, ranging from the use of facial images, top-down body images as well as 3D data. However, numerous highly technical ideas are put forward, most of them are limited to identifying limited number of animals and there needs to be more studies and method development before the camera-based identification system employed in farms.

## Figures and Tables

**Table 1:** Summary of different frequency bands for RFID systems, types of antennae, data speed, and main used cases [23].

Frequency Bands	Antenna	Data and Speed	Read Range	Usage
**Low Frequency (LF) 125 kHz, 134 kHz**	Induction Coil on Ferrite Core, or flat many turns	Low Read Speeds—Small Amount of Data (16 bits)	Short to Medium 3–5 feel	– Access Control – Animal Tagging – Inventory Control – Car Immobilizer
**High Frequency (HF) 13.56 MHz**	Induction Coil flat 3–9 turns	Medium Read Speed Small to Medium amounts of Data	Short 1–3 feet	– Smart Cards – Item or Case level Tagging – Proximity Cards – Vicinity Cards
**Very High Frequency (VHF) 433 MHz—Active Tags**	Internal Custom Design	High Read Speed Large Amount of Data	High 1–1000 feet	– Asset Tracking – Locations – Container Tracking
**Ultra-High Frequency (UHF) 860 MHz—960 MHz**	Single or Double Dipole	High Read Speed Small to Medium Amount of Data	Medium 1–30 feet	– Pallet or Case Level Tagging – DOD and Walmart Mandates
**Microwave Frequency 2.45 GHz and 5.4 GHz**	Single Dipole	High Read Speed Medium Amount of Data	High 1–300 feet	– Container Rail Car – Auto Toll Roads – Pallet Level Tracking

**Table 2:** Commercial availability of different RFID frequency range and their possible use cases [23].

	Low frequency	High Frequency	Ultra High Frequency
**Typical frequency used in livestock**	132.4 kHz	13.56 MHz	866–868 MHz (EU), 902–928 MHz (US)
**Reading range**	0–80 cm	0–1 m	0 cm to 12 m, up to 25 m under certain conditions
**Tags available for livestock**	Yes	Yes, but very limited selection or from other applications	Yes, but limited selection
**Data rate**	4 to 8 kbps	6.7 to 848 kbps	20 to 300 kbps
**Transponder read per second**	<10	>10	>100
**Water interference (proximity of tag)**	No	Low	Strong (absorption)
**Metal interference (reading environment)**	Low	High	High (reflections and interferences)
**Metal interference (proximity of reader antenna)**	High	High	High
